# Exploring real estate blockchain adoption: An empirical study based on an integrated task-technology fit and technology acceptance model

**DOI:** 10.1371/journal.pone.0317993

**Published:** 2025-01-24

**Authors:** Hailan Yang, Zixian Zhang, Chen Jian, Nisar Ahmad

**Affiliations:** 1 School of Business, Shandong Jianzhu University, Jinan, Shandong, China; 2 School of Management, University of Scienceand Technology of China, Hefei, Anhui, China; Mutah University, JORDAN

## Abstract

Although many organizations have adopted blockchain technology (BCT) for efficiency and automation and users’ adoption of BCT is becoming crucial for the companies, few studies have focused on the factors affecting the adoption of BCT in real estate sector. This study aims to assess the factors affecting the adoption of BCT in real estate by the lens of an extended Technology Acceptance Model (TAM) and Task-technology fit (TTF). This current study uses quantitative survey approach. Data were collected from 311 real sector buyers and sellers in China. Partial least square structural equation modeling (PLS-SEM) was used for data analysis. The study’s findings indicate that attitude, perceived usefulness (PU) and data privacy and security (DPS) exerts highest influence in the proposed theoretical model. In addition, the findings also confirmed the significant impact of perceived ease of use (PEOU) on attitude, and TTF impact on PU and PEOU in adoption of BCT. Furthermore, the moderating impact of perceived compatibility (PC) on the relationship between TTF and PEOU was also confirmed. The study has useful practical implications for the real estate buyers and sellers that adoption of BCT improves efficiency, reduce transaction cost incur due to intermediaries, and enhance security system. Furthermore, the study suggests that automation achieved through BCT will facilitate customized agreements and improve process of title transfer.

## Introduction

In the last decade, blockchain technology (BCT) has been implemented in across many contexts, such as hospitality and tourism [1], the healthcare industry [2], open manufacturing [3], education industry [4], and real estate [5]. Although BCT is new, it has reshaped the digital world by offering the latest perspective on business operations’ efficiency, resilience, and security [6]. BCT manages inventory and registers assets [7]. It is a decentralized ledger that accurately records and verifies the transactions between two parties. BCT is a sequence of records categorized into blocks, intricately linked and secure against manipulation by using cryptography. Its inherent structure guarantees resilience against unauthorized modifications within the blocks [8].

The blockchain is handled via a peer-to-peer network that strictly follow protocol for validating new data [9]. Once data is recorded in blocks, any attempt to change it retroactively requires the modification of other blocks, necessitating the support of all parties in the network [10]. In other words, BCT is a distributed database containing transaction records shared among involved participants [11]. BCT is different from traditional ones, where the central subject is tasked with monitoring and tracking responsibility for all data [12]. Crowston et al. [13] explain five stages in real estate transactions: searching, evaluation, property listing, negotiation and execution. These stages involve intermediaries and documentation which delay the process of registration and increase transaction costs. BCT implementation streamlines transactions by improving the registration process in real-time.

Despite technological progress worldwide, transactions in real estate remain slow for many reasons, and the validation process has been considered one of the main issues [14]. Many transaction documents are still relied on paper. The real estate sector relies on manual document validation. Due to abundant documentation in the real estate sector, many mistakes arise, requiring corrections within the registration system [15]. The transaction process in the real estate sector is very inefficient and slow. Higher transaction costs arise because of the time-consuming procedures in registering real estate transactions [16]. Similarly, Fu and Ng [17] found the transaction process slow and inefficient. Therefore, it is necessary to eliminate the bottlenecks in the traditional real estate industry and implement a system that improves its processes and helps the industry to grow.

The economic development of any country is linked with the progress of the real estate market, and its ineffectiveness may result in various transparency issues, increased transaction costs, delays in processes, and biased judgments [18]. BCT can potentially enhance all the inefficiencies within the real estate transaction system. Wang and Kogan [19] argued that BCT could eradicate or reduce fraud and document loss, substantially reducing the lengthy transaction time. In this regard, Worzala and Wyman [20] posits that the aftermath of introducing new technologies into the real estate market is often ambiguous and uncertain. Implementing BCT for transactions involving asset exchange, such as real estate, will substantially diminish the need for third parties [21]. This reduction in intermediaries will lead to reduced transaction times, decreased costs, and enhanced transparency in the process. According to Hoxha and Sadiku [15], the integration of BCT into real estate contracts could result in decreased legal and transaction fees, thus reducing the obstacles for new participants in the system. Tschorsch and Scheuermann [22] argue that BCT upholds transaction transparency and verifies system integrity via smart contracts, eliminating the need for trusted intermediaries. The main parameters for BCT implementations across various domains, including real estate, encompass data privacy, security, and usability [23]. These factors rely on carefully selecting optimal algorithms to guarantee consensus and validity among transaction parties. In addition, in real estate transactions using BCT, all errors must be corrected before finalizing the transaction [24]. The visibility of any changes in the system enhances transparency and trust in real estate transaction records. This process eradicates redundant costs and project real estate dealings. Finally, in the past, brokers and notaries have experienced many cases of counterfeiting and manipulation. However, BCT, functioning as a distributed database housing an expanding list of data items, is significantly resistant to counterfeiting and manipulation [25].

The implementation of Blockchain technology within organizations is still at an early stage, and there is limited research in this area [26]. The current studies in the domain of blockchain examined the application and benefits of blockchain in real estate [27–30]. In recent years, very few studies have focused on adopting BCT [31–34]. Nevertheless, there are some studies on adopting BCT, but its implementation in the real estate sector is limited. Since BCT is still emerging [35], the real estate sector is only beginning to accept it. Hence, additional research is required in the real estate sector to understand blockchain adoption [36], as it will help to reduce insecurity among the buyers and sellers in the real estate sector. In addition, the adoption of BCT in real estate assists its stakeholders and policymakers in making well-informed choices regarding resource allocation and implementing relevant policies [28, 31, 37]. Data privacy and security (DPS) is considered an essential factor in technology driven task [38]. Since real estate sector involves multiple transaction with customer therefore, DPS is high priority of the parties involved in the transaction. DPS is added an antecedent of an integrated TAM and TTF to fill gap in literature and assess the adoption of BCT in real estate sector. Hence, based on TAM and TTF, the current research will addresses following research questions:

RQ1: How does attitude influence the intention to adopt BCT in real estate?
RQ2: How do PEOU, PU and DPS influence the attitude towards BCT in real estate?
RQ3: How does TTF influence the PU and PEOU of adoption of BCT in real estate?
RQ4: What moderating influence does PC exert in the relationship between TTF and PEOU and TTF and PU?

Thus, the current research contributes in blockchain literature by integrating the TTFmodel with the extended TAM and develop a research framework to understand the adoption of BCT in the real estate sector.

### Theoretical background and hypotheses development

**Fig 1:**
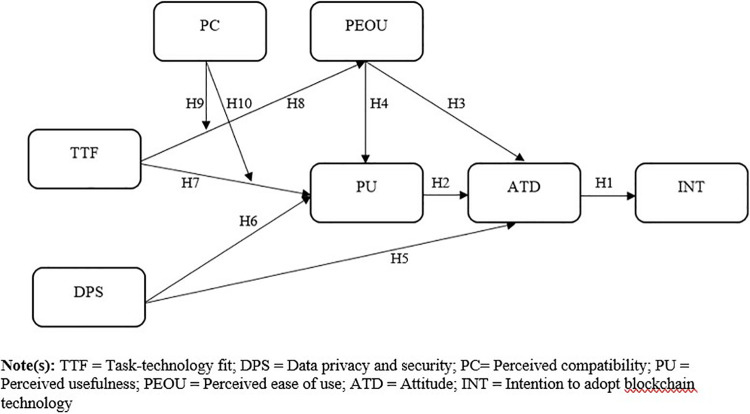
The proposed research model. **Note(s):** TTF = Task-technology fit; DPS = Data privacy and security; PC = Perceived compatibility, PU = Perceived usefulness; PEOU = Perceived ease of use; ATD = Attitude; INT = Intention to adopt blockchain technology.

This study developed a research framework (Fig 1) based on integrated TTF and an extended TAM with data privacy and security as an additional construct. Therefore, the study contains five exogenous constructs that significantly contribute to the intention to adopt blockchain technology in the real estate sector.

### Technology Acceptance Model (TAM)

The Technology Acceptance Model (TAM) [39] has evolved from Ajzen and Fishbein’s [40] Theory of Reasoned Action (TRA). Many IS researchers have employed TAM to assess individual technology adoption [41–43]. Over the years, TAM has proven valuable for accurately predicting the acceptance of technology across many fields, including TV shopping [44], e-learning [45], AI in E-commerce [46], and telemedicine technology [47]. The theory posits that behavioural intention is influenced by two types of responses to the technology features: the affective response and the cognitive response. The affective response is associated with the user’s attitude towards the behaviour. This is related to an individual’s emotional responses, either positive or negative, linked to participating in the anticipated behaviour [48]. Davis [49] explained that PEOU and PU are two primary factors that explain the attitude and intention via specific technology. Davis et al. [50] proposed that TAM can be extended by incorporating additional variables when defining the factors influencing both cognitive and affective responses. The modification in the TAM model is aimed to enhance the model’s suitability for specific technology contexts. In the past, many researchers have extended TAM by including additional variables to predict technology adoption [51–53]. The TAM model was also extended in the perspective of blockchain technology adoption. For example, Kamble [54] has added insecurity and discomfort as external variables in the TAM model. Lou and Li [55] added complexity as an additional variable in TAM to predict the adoption of blockchain technology. Hence, this study incorporated data privacy and security as additional constructs in the TAM model to predict BCT in the real estate sector.

### Attitude

A person’s favourable and unfavourable perspective on engaging in a particular behaviour represents an attitude. Davis et al. [50] argue that within the TAM framework, attitude strongly predicts intention. In the context of BCT adoption, Lou and Li [55] confirmed the strong influence of attitude on intention. Other researchers also confirmed the significant impact of attitude on intention [34, 56, 57]. In the context of real estate, BCT offers peer-to-peer network which enable fast, secure and cost effective transaction [9]. Therefore, this study assumes that attitude will predict intention to adopt BCT in real estate sectors. Hence, it is postulated that:

*H1*: *Attitude will positively influence intention to adopt BCT*.

### Perceived usefulness (PU)

The degree to which a person believes they will get operational or strategic advantages from utilizing a new technology is known as its PU [58]. If a system does not provide perceived benefits, it will likely result in dissatisfaction, negative attitudes, and discontinuance [59]. Prior research indicates that PU is an essential antecedent of BCT adoption [34], which increases customers’ satisfaction and drives them towards adopting BCT [60]. Past research indicates that PU leads to INT across various fields, such as m-commerce [61], the tourism industry [62], innovative companies [56], and the financial service industry [60]. The studies conducted by Albayati et al. [57] and Ullah et al. [63] revealed the positive impact of PU on attitude towards BCT. Esfahbodi et al. [64] found that PU of BCT positively influences E-commerce adoption. Past studies have highlighted the significance of BCT adoption in several industries. Therefore, the adoption of BCT in real estate offers many benefits such as fast transactions, time efficiency, and reduced cost that increase its usefulness among buyers and sellers. Hence, it is postulated that:

*H2*: *PU will positively influence attitude towards BCT*.

### Perceived ease of use (PEOU)

PEOU refers to the degree to which a person believes using a particular technology requires minimal effort [39]. PEOU is associated with the ease of using a particular technology [65]. Past studies found a positive link between PEOU and PU of technology [50, 66] which indicated that reduced effort required for the use of technology enhances its usefulness [67]. Past studies in the technology domain indicated that PEOU is an antecedent of attitude [60, 68]. In the context of BCT adoption, researchers argue that the PEOU of technology leads an individual towards adopting new technology [69, 70]. From the perspective of real estate industry, the adoption of BCT offer smart contract which make process easy for the buyers and sellers and remove intermediaries’ role. Therefore, this study posits that BCT adoption in real estate requires minimum effort which enhance its usefulness and attitude towards its adoption. Hence, it is postulated that:

*H3*: *PEOU will positively influences attitude towards BCT*.
*H4*: *PEOU will positively influences PU of BCT*.

### Data privacy and security (DPS)

In the real estate sector, documents establish identity and are considered necessary in legal matters [35]. Therefore, DPS are essential for businesses. BCT facilitates peer-to-peer interactions, minimizing the threat of gathering data from third parties, thereby maintaining the parties’ anonymity without disclosing their identities [71]. This offers significant online protection, enabling customers to consciously use and share their information while maintaining the highest level of privacy [72]. The study conducted by Lallmahamood [73] revealed that DPS in the TAM model significantly influence the usefulness of the technology. Similarly, Esfahbodi et al. [64] indicate the significant influence of DPS on the adoption of BCT. In the context of real estate, BCT implementation facilitate peer-to-peer interactions and enhance parties’ privacy. Therefore, it can be inferred that DPS is an essential factor from buyers and sellers perspective as it will significantly influence BCT usefulness and develop a positive attitude. Hence, it is postulated that:

*H5*: *Data privacy and security will positively influence attitude towards BCT*.
*H6*: *Data privacy and security will positively influence PU of BCT*.

### Technology-task fit (TTF) model

Information technology (IT) can facilitate a task by aligning the technology with the task’s requirements [74]. Goodhue and Thompson [75] proposed the TTF model, which focuses on matching the technology to a task [76]. The TTF model posits that the acceptance of technology depends on its fit with the requirements of a specific task [77], and it extends the TAM by taking into account how the task influences intention [78]. TTF refers to how effectively a technology assists an individual in completing various tasks. Tasks include individuals’ efforts to convert inputs into outputs, and technologies are considered the best tools to accomplish these tasks [79, 80]. Since TTF’s creation, it has been implemented across various information systems, including information and communication technologies adoption [81], AI-based Chatbot in the banking sector [82], augmented reality app in the retailing sector [83], and SME’s blockchain adoption [84]. Although TTF has been used in various sectors, limited research has been conducted in the context of the real estate sector. To understand whether a good TTF will influence blockchain technology adoption in the real estate sector. Therefore, the current study integrates TTF with TAM to predict the adoption of BCT in the real estate sector.

TTF suggests that users will adopt the technology if it fulfils the task’s requirements [85] in a more efficient manner and at reduced cost [86]. Since the introduction of TTF by Goodhue and Thompson [75], many researchers have employed it to predict technology adoption [87–89]. Dishaw and Strong [90] integrated TTF with the TAM model and confirmed the positive influence of TTP on PU. Wu and Chen [77] revealed that TTF has a positive and significant influence on the PU of MOOCs. Foroughi et al. [58] found that TTF positively influenced PU and PEOU of food delivery apps. Howard and Hair Jr [91] integrated TTF with TAM and found that the TTF significantly influenced the adoption of technology by PU and PEOU in myriad industries. Therefore, the current study proposes that TTF will positively influence the PU and PEOU of blockchain technology in the real estate sector.

Hence, it is postulated that:

*H7*: *TTF will positively influence PU of BCT*.
*H8*: *TTF will positively influence PEOU of BCT*.

### Perceived compatibility (PC)

Compatibility represents the degree to which an innovation or technology matches the customers’ requirements [92, 93]. Taylor and Todd [94] posited that if the technology features highly match users’ requirements, the chances of technology acceptance will be higher. Researchers have frequently used PCs to assess their impact on the intention to adopt IT [95]. For example, the study conducted by Lai and Chen [96] revealed that the adoption of teaching blogs was significantly influenced by PC. Chung et al. [97] argued that PC technology increases job performance. Suggested that PC strengthen the relationship between e-learning and academic performance [98]. Huang and Yu [99] confirmed that PC significantly moderates the relationship between PU and green product satisfaction. Research also indicates that PC significantly influences mobile banking adoption’s PU and PEOU [100]. BCT is compatible in real estate as it streamlines transactions by offering timely, secure and cost effective solutions to buyers and sellers. It also simplifies the ownership transfer and verification through smart contracts. This study assumes that BCT is compatible in real estate industry, therefore, PC will strengthen TTF relationship with PU and PEOU. Hence, it is postulated that:

*H9*: *PC will moderate the relationships between TTF and PEOU*, *specifically customers with higher level of PC will have a strong relationship between TTF and PEOU*.
*H10*: *PC will moderate the relationships between TTF and PU*, *specifically customers with higher level of PC will have a strong relationship between TTF and PU*.

## Methodology

### Research instrument

This study used a questionnaire based on established scales from past studies, which had been pre-validated and achieved significant reliability and validity. The questionnaire layout consists of three parts. The first part was related to the purpose of the study. The second part of the questionnaire was related to participants’ informed consent. The third part contained the variables’ names and their items. A seven-point Likert scale ranging from strong disagree to agree strongly was used to measure the items. PEOU and PU scales were adopted from the scales developed by Davis [39] and Kamble et al. [34]. PEOU and PU were measured using four scales for each item. The study adopted five items from the Kamble et al. [34] study to measure the ATD scale. DPS was measured using a four-item scale developed by Esfahbodi et al. [64] and Lallmahamood [73]. Task-technology fit was measured using a four-item scale developed by Dhiman and Jamwal [87]. Intention to adopt BCT was measured using a five-point scale developed by Ruangkanjanases et al. [101]. PC was measured using a three-item scale developed by Huang and Yu [99]. A pre-test of the instrument was conducted with the help of three academics to evaluate content and face validity. The instrument layout and item changes have been incorporated based on participants’ recommendations. Two professionals translated the questionnaire from English to Chinese and then from Chinese to English. Then, another expert compared the translated items with the original items to confirm that the meaning remained unchanged. The final version of the questionnaire was validated through pilot testing involving 44 participants. The results of the pilot test confirmed the reliability of the constructs.

### Data collection

This study utilized a quantitative deductive approach, and a field survey was conducted to collect the data from real estate buyers and sellers in six Chinese cities: Jinan, Liaocheng, Dezhou, Xingtai, Puyang, and Zibo. The data was collected using a purposive sampling technique as this approach enable researchers to collect data from the participants who can fulfill the research objectives. To minimize the retrieval biases, the researcher provides guidelines regarding the study scope and contact number of the corresponding author for further information [102]. After introducing the research topic, the questionnaire was distributed to the participants. A total of 518 questionnaires were distributed to the study participants. Most participants agreed to participate in the research and filled out 344 questionnaires. Out of the filled questionnaire, 311 were deemed suitable for further analysis. The details of participants’ demographics are shown in Table 1.

**Table 1:** Demographics.

Demographic variables	Classification	Frequency	Percentage
Gender	Male	213	68.5%
	Female	98	31.5%
Roles	Buyer	189	68.5%
	Seller	122	31.5%
Age	18 to 25 years	67	21.5%
	26 to 33 years	110	35.4%
	34 to 41 years	66	21.2%
	42 to 49 years	40	12.9%
	Above 49 years	28	9.0%
Experience	Below 5 years	13	4.2%
	6 to 10 years	119	38.3%
	11 to 15 years	134	43.1%
	Above 15 years	45	14.5%
Family Income	Below 50000 PKR	63	20.3%
	50001 PKR to 75000 PKR	124	39.9%
	75001 PKR to 100000 PKR	74	23.8%
	100001 PKR to 125000 PKR	18	5.8%
	125001 PKR to 150000 PKR	18	5.8%
	More than 150000 PKR	14	4.5%

### Data analysis

“The partial least square structural equation modelling”- (PLS-SEM) was used to analyse data. It is a flexible approach that can be used across settings with small sample sizes and lenient distribution requirements compared to other modelling techniques [103]. This study used SmartPLS 4.1.0.0 [104] to analyse data. The study followed the standard guideline by employing a two-step approach [105]. In the first step, measurement was evaluated by assessing convergent and discriminant validities. In the second step, the study evaluated the structural model.

## Results

### Preliminary data analysis

Preliminary analysis was conducted to ensure that it is suitable for further analysis. First, the study checked for non-response bias by evaluating early and late responses [106]. The results of the paired samples test indicate that there is no significant difference in early and late responses. Then, common method bias (CMB) was performed. Harman’s single-factor test was used to evaluate the variance explained by the single factor. The results show that a single factor explained 32.66% of the data, which is less than 50%, confirming that CMB is not an issue [107]. In addition, variance inflation factor (VIF) values were checked to assess multi-collinearity. The results (see Table 2) indicate that VIF values range from 1.371 to 2.740, which are < 3, confirming the absence of multi-collinearity [108]. These findings suggest that predictors are not correlated which enhance coefficient reliability to predict outcomes more accurately.

**Table 2:** Measurement model assessment.

Constructs/items	FL	VIF	(α)	CR	AVE	Mean	SD
**Task-technology fit**			0.780	0.872	0.694	5.85	0.76
TTF1: The use blockchain technology fits well with real estate-related tasks and needs.	0.842	1.616					
TTF2: The use of blockchain technology fits well with the way I like to execute my real estate-related tasks.	0.800	1.532					
TTF4: The use of blockchain technology fits well with all aspects of managing real estate.	0.857	1.761					
**Data privacy and security**			0.840	0.889	0.669	5.55	0.96
DPS1: Customer data can be protected by the use of blockchain technology.	0.743	2.139					
DPS2: Blockchain technology protects data against unauthorized access.	0.862	2.740					
DPS3: Blockchain technology protects from customers’ transactional information, preventing changes during real estate transaction	0.782	1.469					
DPS4: Blockchain technology employs appropriate security measures to safeguard customers’ personal data	0.876	2.679					
**Perceived ease of use**			0.792	0.878	0.706	5.59	1.05
PEOU1: I think the features blockchain technology will be easy to use.	0.807	1.564					
PEOU2: I feel it will be easy for me to perform tasks using Blockchain technology.	0.895	1.992					
PEOU4: I think blockchain technology will be more easy to use compared to the conventional practices of managing real estate task.	0.816	1.678					
**Perceived usefulness**			0.799	0.869	0.626	4.07	1.04
PU1: Blockchain technology will help to reduce the transaction delays.	0.814	1.668					
PU2: Blockchain technology will help to improve real estate performance.	0.700	1.371					
PU3: Blockchain technology will help to improve real estate efficiency.	0.818	1.971					
PU4; Blockchain technology will help to improve real estate effectiveness.	0.826	2.002					
**Attitude**			0.745	0.838	0.565	5.13	0.69
ATD1: I think it is needed to use blockchain technology in real estate.	0.761	1.389					
ATD3: I think using blochchain technology is a good idea.	0.802	1.713					
ATD4: I have a favorable attitude toward blockchain technology.	0.670	1.511					
ATD5: I will be happy if my company implements block chain technology.	0.767	1.477					
**Intention**			0.854	0.895	0.631	5.43	1.04
INT1: I decide to use Blockchain technology in future.	0.838	2.120					
INT2: I will continuously use Blockchain technology.	0.832	2.060					
INT3: I will use different features of Blockchain technology.	0.781	1.824					
INT4: I intend to use Blockchain technology in different areas of my life.	0.742	1.674					
INT5: The use of blockchain technology will make life easier.	0.775	1.788					
**Perceived compatibility**			0.755	0.860	0.672	6.07	0.60
PC1: Blockchain technology is compatible with my office task.	0.791	1.387					
PC2: Using blockchain technology fits well with the way I like to manage my work.	0.836	1.650					
PC3: Using blockchain technology to conduct real estate transactions fits into my work profile.	0.831	1.617					

**Note(s):** FL = Factor loading; (α) = Cronbach Alpha; CR = composite reliability; AVE = average variance extracted; VIF = variance inflation factor, SD = Standard deviation.

## Measurement model

Following the researchers’ guidelines [103], first, the study evaluated the measurement model by assessing reliability and validity. First, the loading of measurement items (indicators) was assessed. A loading score of ≥ 0.708 signifies the minimum acceptable threshold value. Table 2 shows that all item’s loading scores are greater ≥ than 0.708 except for PU2 and ATD4. Sarstedt et al. [107] suggested removing the item with loading between 0.40 and 0.70 if it increases the composite reliability values (CR) and average variance extracted (AVE). Items should be retained with lower loading if it contributes to validity. Following these suggestions, the CR and AVE values were assessed after removal and addition of PU2 and ATD4 items. The results indicate no substantial difference in the CR and AVE values. Therefore, the study included these items for further analysis. Second, the study assessed Cronbach’s Alpha (α) and CR for internal consistency. It is recommended that (α) and CR values should be minimum 0.70 and not exceeds 0.95 [108]. CR and (α) values are above 0.70 and below 0.90 meeting internal consistency requirement (see Table 2). Third, the study assessed convergent validity by evaluating AVE. The minimum threshold value for the AVE is 0.50. Table 2 shows that all constructs have greater than 0.50 AVE value, confirming the presence of convergent validity. Finally, the discriminant validity was assessed using Fornell and Larcker’s and Heterotrait–Mono trait Ratio (HTMT) criterion. The square root of the AVE for a construct must exceeds its correlation with any other construct. This requirement is satisfied (see Table 3), indicating the presence of discriminant validity [109]. In addition, HTMT results (see Table 4) indicate < 0.90 HTMT value [110], endorsing the findings established through Fornell and Larcker’s criterion.

**Table 3:** Fornell-Larcker criterion.

Constructs	1	2	3	4	5	6	7
Attitude	0.751						
Data privacy and security	0.641	0.818					
Intention	0.623	0.723	0.794				
Perceived compatibility	0.484	0.360	0.301	0.819			
Perceived ese of use	0.597	0.485	0.516	0.389	0.840		
Perceived usefulness	0.687	0.500	0.581	0.381	0.512	0.791	
Task-technology fit	0.607	0.451	0.320	0.447	0.354	0.497	0.833

**Table 4:** Heterotrait-monotrait ratio (HTMT) criterion.

Constructs	1	2	3	4	5	6	7
Attitude							
Data privacy and security	0.750						
Intention	0.739	0.799					
Perceived compatibility	0.643	0.441	0.368				
Perceived ese of use	0.764	0.561	0.620	0.499			
Perceived usefulness	0.895	0.555	0.689	0.487	0.636		
Task-technology fit	0.820	0.562	0.379	0.580	0.442	0.627	

### Inner model predictive power

Inner model predictive power demonstrates how well the proposed model predicts the relationships. Two statistical measures are used to assess the predictive power of the inner model: coefficient of determination (R2) and predictive relevance (Q2). The R2 is the variance explained by exogenous on endogenous constructs. R2 values of endogenous constructs INT, attitude, PEOU, and PU are 38.8%, 62.2%, 20.7% and 41.4%, respectively, which show that exogenous constructs have explained moderate to substantial variance in the proposed model. Q2 is the predictive accuracy of the proposed model. Q2 value above 0 indicates predictive accuracy. Q2 values of endogenous constructs INT, attitude, PEOU, and PU are 23.7%, 34.1%, 13.3% and 24.9%, respectively, which show moderate to high predictive accuracy of the model.

## Structural model

**Fig 2:**
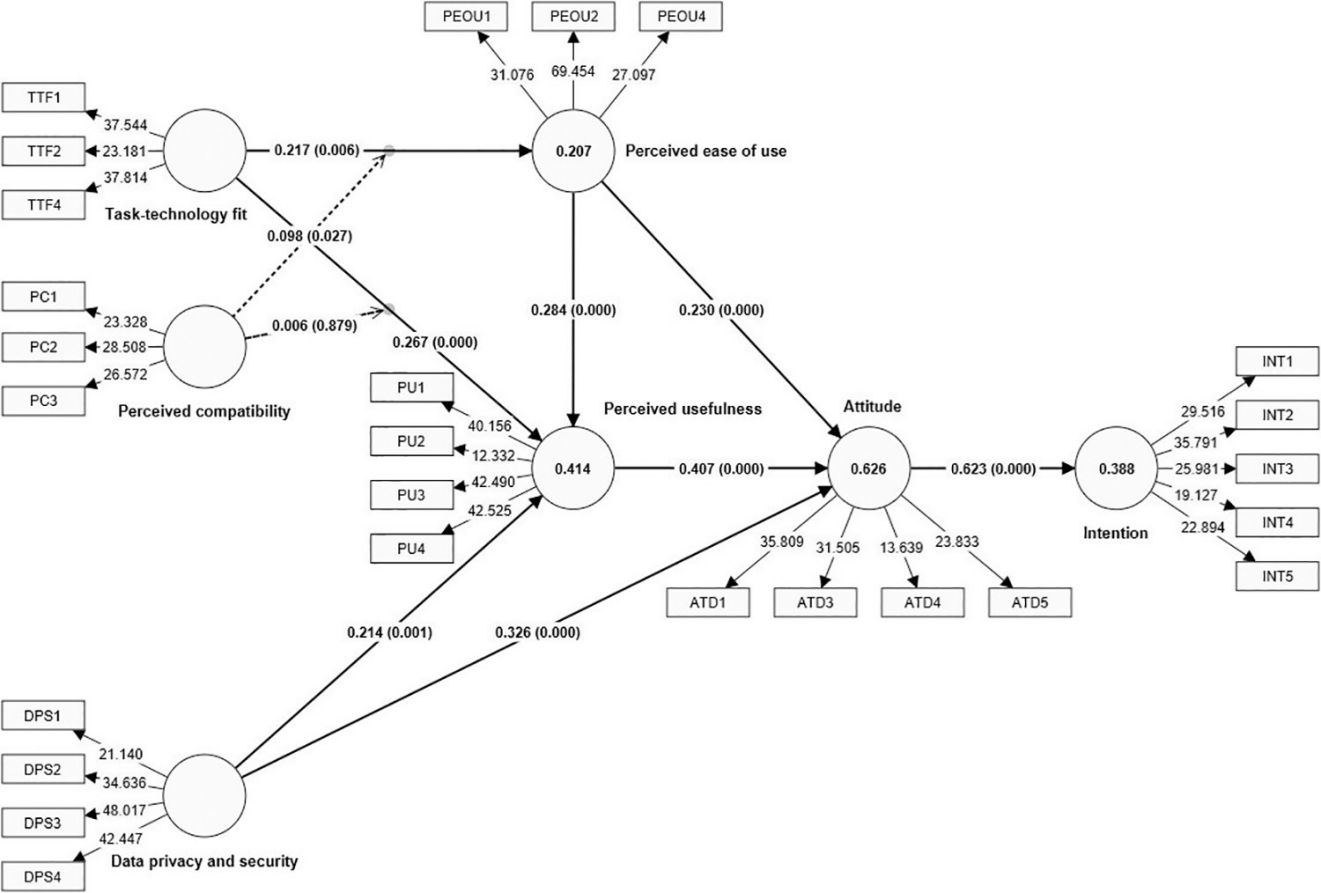
Structural model.

This study employed 5000 bootstraps to assess the research model. There were ten hypotheses: eight were direct effects, and two were moderating effects. Examining the structural model includes the path coefficients (β), p value, and t values. Fig 2 and Table 5 indicate the relationship among constructs, path coefficients (β), p values, and t values. The results show positive significant impact of attitude on INT (H1: β = 0.144, t = 12.267, p < 0.01); positive significant impact of PU on attitude (H2: β = 0.144, t = 12.267, p < 0.01); positive significant impact of PEOU on attitude (H3: β = 0.144, t = 12.267, p < 0.01); positive significant impact of PEOU on PU (H4: β = 0.144, t = 12.267, p < 0.01); positive significant impact of DPS on attitude (H5: β = 0.144, t = 12.267, p < 0.01); positive significant impact of DPS on PU (H6: β = 0.144, t = 12.267, p < 0.01); positive significant impact of TTF on PU (H7: β = 0.144, t = 12.267, p < 0.01); positive significant impact of TTF on PEOU (H8: β = 0.144, t = 12.267, p < 0.01).

**Table 5:** Hypotheses testing.

Hypotheses	Path coefficient	T value	P values	Decision
H1: Attitude -> Intention	0.623	17.407	0.000	Supported
H2: Perceived usefulness -> Attitude	0.407	7.707	0.000	Supported
H3: Perceived ease of use -> Attitude	0.230	3.892	0.000	Supported
H4: Perceived ease of use -> Perceived usefulness	0.284	5.518	0.000	Supported
H5: Data privacy and security -> Attitude	0.326	6.541	0.000	Supported
H6: Data privacy and security -> Perceived usefulness	0.214	3.462	0.001	Supported
H7: Task-technology fit -> Perceived usefulness	0.267	4.429	0.000	Supported
H8: Task-technology fit -> Perceived ease of use	0.217	2.773	0.006	Supported
H9: Perceived compatibility x Task-technology fit -> Perceived ease of use	0.098	2.207	0.027	Supported
H10: Perceived compatibility x Task-technology fit -> Perceived usefulness	0.006	0.153	0.879	Not supported

**Note(s):** Path coefficients are significant at 0.001 and 0.005 levels.

## Analysis of moderating effect

**Fig 3:**
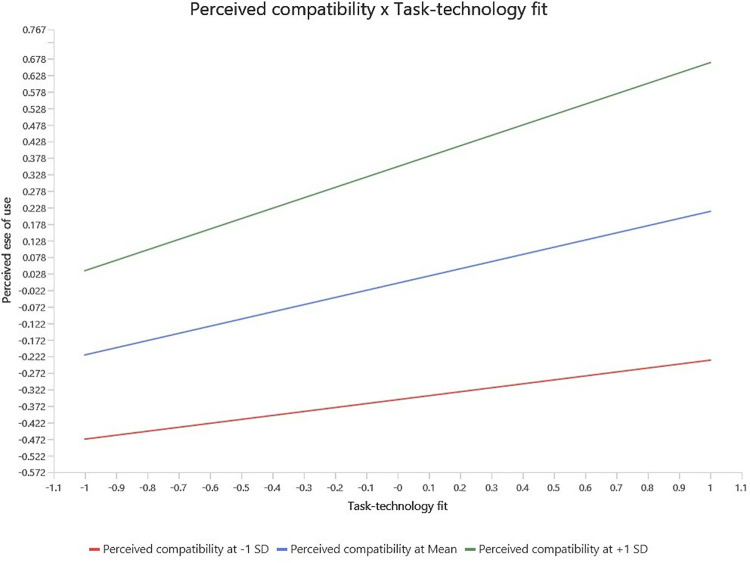
Moderating effect of PC on TTF and PEOU relationship.

**Fig 4:**
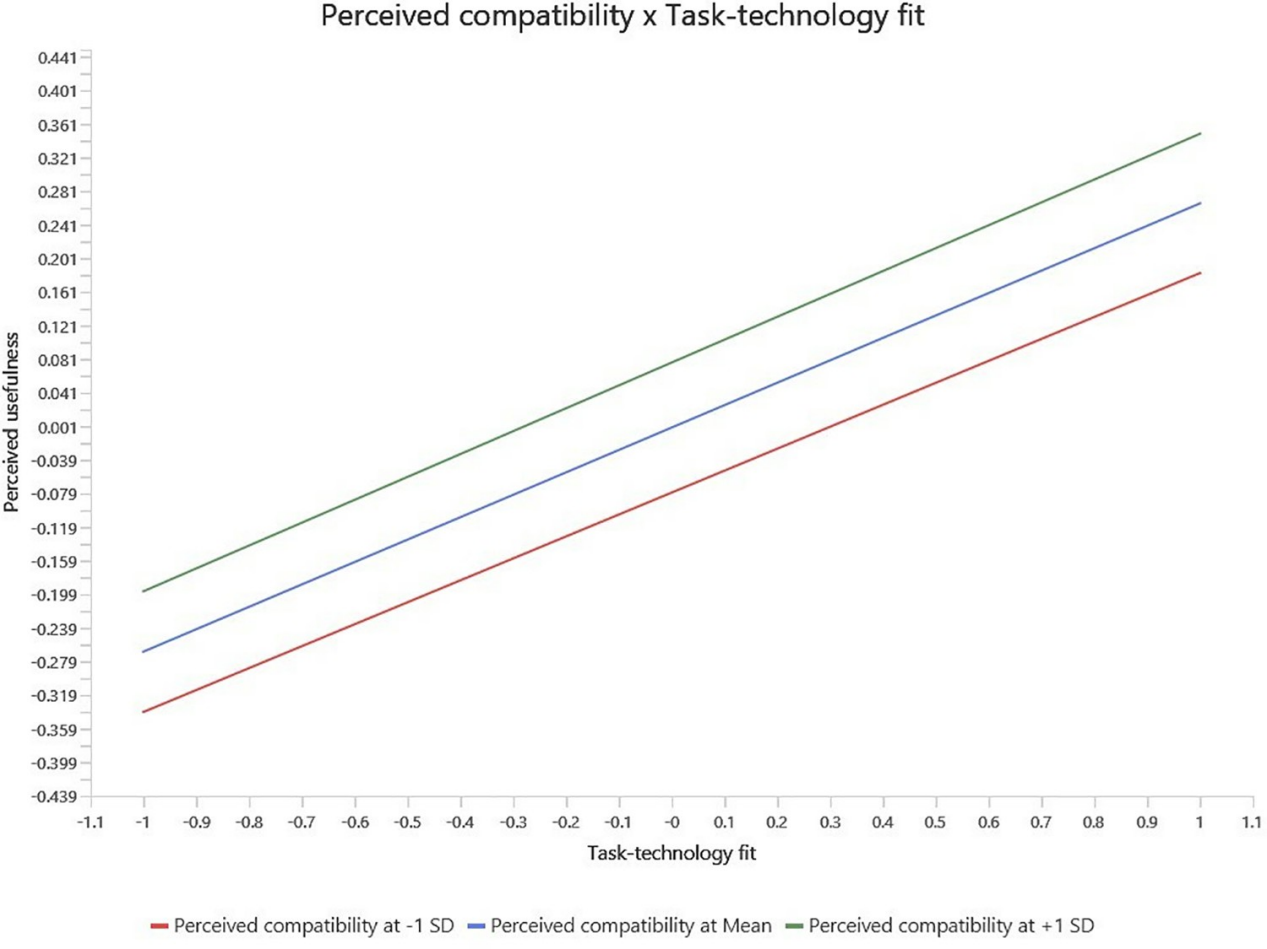
Moderating effect of PC on TTF and PU relationship.

In order to examine the moderating effect, the study utilized product indicator method (Sarstedt et al., 2021). The moderating analysis was done using SmartPLS 4.1.0.0 version. First, moderator PC was added as moderator on the relationship between TTF and PEOU, and the relationship between TTF and PU (see Fig 2). Then, bootstrapping function was utilized to determine the path significance. The results of the moderating analysis show that PC significantly moderates the relationship between TTF and PEOU. However, the positive moderating effect of PC on the relationship between TFF and PU is insignificant. To further elaborate the moderating findings, the slope analysis (see Fig 3) show that if PC is high, the relationship between TTF and PEOU becomes stronger and vice versa. In case of the moderating effect of PC on the relationship between TTF and PU, the slope analysis (see Fig 4) show that if PC is high, it has no effect on the relationship between TTF and PU.

## Discussion

This research evaluated the real estate buyers’ and sellers’ INT to adopt BCT using an integrated TAM and TTF model. The study also assessed the moderating effect of PC on the relationship between TTF and POEU, as well as the relationship between TTF and PU. The study’s findings revealed the significant impact of attitude on INT to BCT. The findings are consistent with previous studies [34, 55, 57]. Buyers’ and sellers’ positive attitudes and INT to adopt BCT signify the relevance of BCT in the real estate sector. They are willing to adopt BCT because it streamlines real estate transactions and improves efficiency. In past, researchers highlighted that traditional real estate registration system is inefficient [17] and contain lots of error [15] that must be modified with the use of advanced technologies. Therefore, BCT adoption is essential to improve registration in real estate and enhance its performance. Next, the findings show a positive significant impact of PU on attitude towards BCT adoption, consistent with past studies [34, 63]. It indicates the usefulness of BCT in the real estate sector. BCT reduces the administrative tasks of the employees in the real estate sector and offers robust technology to safeguard contracts with partners. In addition, BCT facilitates property management through smart contracts functioned by programming codes that operate automatically when the specific requirement is fulfilled. The findings also confirmed the positive significant impact of PEOU on attitude towards BCT. The findings are consistent with the studies of Kumari and Devi [60] and Liesa-Orús et al. [68]. It shows that users might have previous experience using blockchain technology, affecting their perception of the ease of using the BCT. Another possible reason could be that the BCT user-friendly interface and users’ technology learning curve increase the PEOU of BCT. Regarding the positive influence of DPS on the attitude and PU of BCT, the study’s findings confirmed the proposed positive relationship between them. These findings are consistent with past studies that explain the significance of privacy shaping customers’ attitudes towards technology [64, 72]. The findings of Lallmahamood [73] indicate that DPS is an essential feature of technology usefulness. Furthermore, the adoption of BCT eliminate the elimination of third party involvement enhances its security and provide more relief to the buyers and seller during transaction [21]. Additionally, BCT adoption ensures decentralized transaction which is encrypted and attached with previous one to protect data from tempering [30]. These features of BCT reduces online fraud and human errors. Based on this study’s findings, BCT protects users from property title theft and other fraudulent activities that enhance the usefulness of BTC in the real estate sector. In addition, the transparency offered by the BCT provides an edge to companies and attracts more customers, which enhances the PU of BCT in the real estate sector.

Furthermore, the findings confirmed the significant impact of TTFs on PU, which aligns with the previous researchers’ work [58, 77]. The positive impact of TTF on PU indicates that BCT minimizes the risk associated with manual work by the individual. It also facilitates peer-to-peer transactions and stakeholders’ participation in real estate transactions enabled by blockchain technology. The findings also support the significant positive impact of TTF on PEOU, which is consistent with the findings of past studies [58, 91]. These findings indicate that BCT includes a user-friendly interface, provides clear instructions, and makes users feel it is easy to use BCT. It also highlights that BCT does not require troublesome processes to execute the tasks and is easy to operate in the real estate sector. Next, the results indicate the significant moderating effect of PC on the relationship between TTF and PEOU. Past studies have also shown that the match between different features of technology and users’ experience leads to increased technology adoption [94, 96]. When people in the real estate sector feel that BCT is compatible with their real estate objectives and daily activities, its relevance increases, and they think it is easy to use. PC of BCT in the real estate sector increases people’s confidence in directing the technology, which minimizes the perception of difficulty in using BCT. However, the results indicate the insignificant moderating effect of PC on the relationship between TTF and PU. The possible reason for this insignificant relationship may be due to BCT’s perceived inefficiency in the real estate sector. Users might feel BCT will not help them achieve transparency, automation, and security in real estate transactions. PC is an individual belief for technology efficacy which is less effective in the real estate context because the usefulness of BCT precedes personal belief because BCT directly provide solutions to industry-specific issues such as transparency, privacy and automation. Therefore, educating real estate professionals about BCT benefits such as security and automation is essential. The knowledge of these benefits enhances real estate professional competency, and improve technology diffusion and dissemination of information to customers for greater adoptability.

## Conclusion

BCT can change the dynamics of the real estate sector by ensuring modern, secure, and transparent property transactions. Therefore, this study examined the real estate buyer and seller’s INT to adopt BCT. It integrated TTF with extended TAM by incorporating DPS in the research framework. The quantitative survey method was employed, and participants were approached in six Chinese cities using purposive sampling. The study used SmartPLS 4.1.0.0 for SEM analysis and SPSS 26 for the descriptive analysis. The study’s results indicate that the proposed research framework significantly influences buyer and seller’s INT to adopt BCT in the real estate sector. The study’s findings show that attitude, PU, PEOU, TTF, and DPS are highly significant in INT’s adoption of BCT. Further, the findings confirmed the significant moderating effect of PC on the relationship between TTF and PEOU of BCT.

## Theoretical implications

Theoretically, the study has contributed to the literature on TAM and TTF in the context of BCT adoption in the real estate industry. The findings derived from an integrated TAM and TTF confirmed stakeholders’ intention to adopt BCT in the real estate industry. This is one of the first studies that explored BCT adoption in a real estate context by exploring the nexus among personal and technology related factors using an integrated TAM and TTF, thus enriching the literature on BCT. Further, this In the context of BCT adoption in real estate, DPS is an essential factor, therefore, this study added DPS as an antecedent of TAM to predict BCT adoption. In the past, the direct effect of DPS on attitude and PU was not explored in the adoption of BCT. This study confirmed that DPS is the direct antecedent of attitude and PU’s adoption of the real estate sector. These relationships signify that DPS is a critical factor in adopting BCT in the real estate sector because users’ feel that it is relevant and meet the needs of real estate sector. Further, to the best of the researcher’s knowledge, this is the first study that explored the moderating effect of PC on the relationship between TTF and PEOU in the context of real estate. The findings confirmed that PC plays a vital role in the TTF and PEOU nexus.

## Practical implications

There are some practical implications of this study. First, the BCT is facilitate the buyer and seller through peer-to-peer transaction. Therefore, the findings indicate strong impact of attitude on INT to adopt BCT. It also helps to maintain the records and preserve data for long-time period increases users’ efficiency and reducing the maintaining costs. PEOU positively influences the BCT adoption indicating the significance of ease of using the technology for the accessing and verification of the property. Secondly, the stakeholders in real estate can obtain the holistic benefits of BCT within the real estate eco-system which is more effective in BCT adoption rather than merely focusing on its features. As the study results indicate that BCT is well-fitted within the real estate sector, BCT training related to automation (stored codes execute automatically once the predetermined conditions are met), public accessibility, tracking assets, enabling property tokenization (eliminating the bank financing and timeline limitation for investing in properties) will increase its adoption in the real estate sector. The findings also indicate the usefulness of BCT in real estate due to its tailored solutions to meet specific needs. For example, real estate companies can collaborate with IT experts or developers to design solutions that address industry challenges. The experts and industry collaboration for the blockchain application process will improve technology relevancy and increase its adoption in real estate. In addition, results indicate that BCT compatibility in real estate makes it easier for the buyer and seller to communicate, eliminating the roles of intermediaries such as banks, lawyers and brokers. These intermediaries increase costs, slow processes, and require more documentation. Implementing BCT will remove intermediaries’ roles, connect buyers and sellers and make the process faster. Additionally, the findings suggest that stakeholders feel that BCT is well-suited for real estate operations and it is easy to use which increases its adoption. However, the insignificant influence of PC on the relationships between TTF and PU suggests that proper training is required to improve professional understanding of BCT benefits in the real estate industry. BCT training can be theoretical and hands-on to understand the operational benefits of BCT. Theoretically, the real-world case studies are best to depict BCT’s significance in the real estate industry. Practical training includes workshops that demonstrate the implementation of smart contracts and the verification of documents. Furthermore, professionals should be encouraged to acquire professional courses on BCT to understand the complexities and challenges related to technology implementation and understanding its benefits for business growth.

## Limitations and future research

Although this study provides a new perspective to understand BCT adoption in real estate sector, it is necessary to consider some limitations of the study. The study predictors explained the substantial variance to adopt BTC in real estate sector. For comprehensively understanding the adoption of BCT in real estate sector, the influence of various factors including technology related and individual may be incorporated to increase the explanatory power of the model. The data has been collected from six adjacent cities from the Shandong, Henan, and Hebei provinces. Future research may consider collecting data from the majority of the provinces by including more cities for greater understand of BCT adoption in real estate sector. Furthermore, this study is cross sectional, so the researcher may not be able to capture the actual behavior of the participants. Hence, a longitudinal research is required to understand the actual behavior of the participants over the period of time.
